# An Antidote to the Imager's Fallacy, or How to Identify Brain Areas That Are in Limbo

**DOI:** 10.1371/journal.pone.0115700

**Published:** 2014-12-29

**Authors:** Gilles de Hollander, Eric-Jan Wagenmakers, Lourens Waldorp, Birte Forstmann

**Affiliations:** 1 University of Amsterdam, Amsterdam Brain and Cognition (ABC), Amsterdam, the Netherlands; 2 University of Amsterdam, Department of Psychology, Amsterdam, The Netherlands; Universiteit Gent, Belgium

## Abstract

Traditionally, fMRI data are analyzed using statistical parametric mapping approaches. Regardless of the precise thresholding procedure, these approaches ultimately divide the brain in regions that do or do not differ significantly across experimental conditions. This binary classification scheme fosters the so-called imager's fallacy, where researchers prematurely conclude that region A is selectively involved in a certain cognitive task because activity in that region reaches statistical significance and activity in region B does not. For such a conclusion to be statistically valid, however, a test on the differences in activation across these two regions is required. Here we propose a simple GLM-based method that defines an “in-between” category of brain regions that are neither significantly active nor inactive, but rather “in limbo”. For regions that are in limbo, the activation pattern is inconclusive: it does not differ significantly from baseline, but neither does it differ significantly from regions that do show significant changes from baseline. This pattern indicates that measurement was insufficiently precise. By directly testing differences in activation, our procedure helps reduce the impact of the imager's fallacy. The method is illustrated using concrete examples.

## Introduction

### The Imager's Fallacy

In this paper, we introduce an approach to test and describe effects across conditions and regions in functional Magnetic Resonance Imaging (fMRI) data. This approach explicitly marks regions in which activation is in an in-between state of uncertainty, statistically differing neither from baseline nor from regions that are themselves significantly active. We label such regions “in limbo”.

Our approach facilitates a more cautious and honest interpretation of Statistical Parametric Maps (SPMs), as these maps are prone to the so-called imager's fallacy [1], [3]. Due to the nature of fMRI contrasts, usually represented as color-coded “brain maps”, it is tempting to draw explicit or implicit conclusions such as in the following (fictional) statement: “The SPM shows that that activation in the primary motor cortex (M1) was significantly increased, whereas activation in the substantia nigra (SN) was not; hence, we conclude that M1 is selectively involved in the task at hand.” Such conclusions, however, are premature: for instance, it may be that due to higher levels of noise in the fMRI signal from subcortical areas [4], the variance in the SN was considerably higher than that in the primary motor cortex, preventing an effect that actually exists to reach statistical threshold. Such Type II errors are far from unlikely in fMRI studies, which are usually underpowered [5].

Statements about selective activation cannot be made without proper statistical testing. To test for selective activation, it is required to consider the interaction between region and condition. In other words, one needs to test whether the two regions of interest show a different pattern of activation [2], [6]. Although some neuroimaging studies with predefined regions of interest test for this region x condition interaction (e.g. [7], [8]), studies with a whole-brain analysis almost always omit this step, possibly drawing false inferences because of the imager's fallacy.

**Figure 1:**
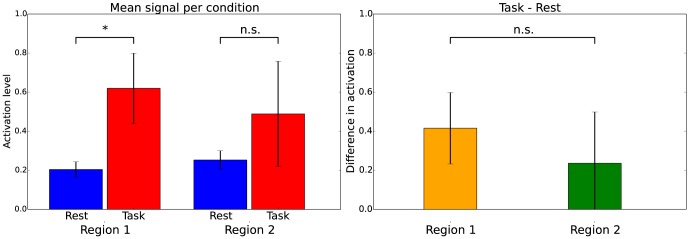
Illustration of the imager's fallacy: during the task condition, region 1 shows significantly increased activation as compared to the rest condition. Region 2 does not show significantly increased activation. One could be inclined to conclude that region 1 is selectively involved in the task. However, when the sizes of these contrasts are tested against each other, the size of the contrast in region 1 does not significantly differ from the size of the contrast in region 2.

Fig. 1 illustrates the imager's fallacy: in this example, one region shows significantly more activation during a task than during rest and a second region does not. However, the difference in activation between rest- and task-condition in almost identical in these two regions and selective activation of the first region cannot be concluded.

In this paper, we present a whole-brain analysis that explicitly identifies regions which are at risk for the imager's fallacy, because their experimental effect is not significantly different from “significantly activated” regions: these regions are therefore in limbo, and their true status awaits further investigation.

In almost all standard fMRI-analysis approaches, the brain is ultimately divided in regions that show a significant difference in BOLD-response across conditions and regions that do not, thereby inviting the imager's fallacy. In contrast, our in limbo approach further subdivides the non-significantly activated regions in two categories: regions that differ from those that show significant activation and regions that do not. The latter regions are “in limbo”: they differ neither from baseline nor from regions that do differ from baseline.

## Methods

### The In Limbo Approach

**Figure 2:**
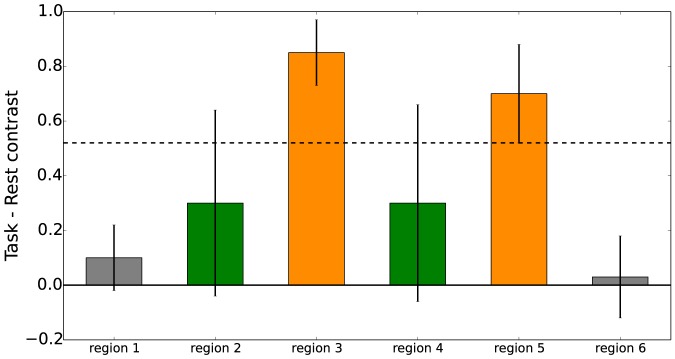
An example of the in limbo approach. Shown are hypothetical contrast sizes and confidence intervals for 6 regions (these could be individual voxels). For region 1 and 6 there is no significant effect: zero falls well within the confidence intervals of the contrast size of in these regions. For region 3 and 5, there is a clear effect: the “task rest” contrast differs significantly from zero. For region 2 and 4 the situation is more complicated: the confidence interval of the “task rest” contrast still contains 0, so one is unable to reject the null hypothesis. However, the size of the contrast is not significantly different from that of the contrast in region 5. Regions 2 and 4 are “in limbo”: they differ neither from baseline, nor from least-significantly activated region. Legend: Orange areas are significantly activated, green areas are in limbo gray areas are significantly less activated than significant regions.

**Figure 3:**
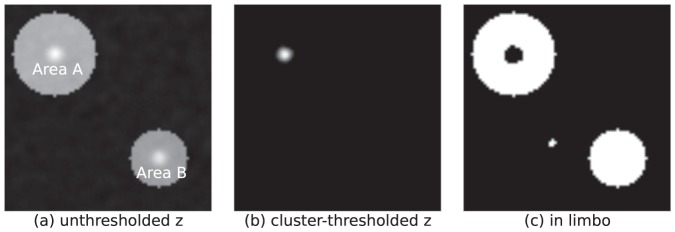
Simulation results for an in limbo analysis of a single subject. In a standard fMRI analysis, some region, region A, can be identified as significantly more activated during a specific condition. For another region, region B, this effect is not significant. However, region B also does not differ significantly from region A. Hence, it is incorrect to conclude that region A is selectively activated; instead, it is appropriate to conclude that region A is activated, and region B is in limbo. See also section *Simulation Studies*.

**Figure 4:**
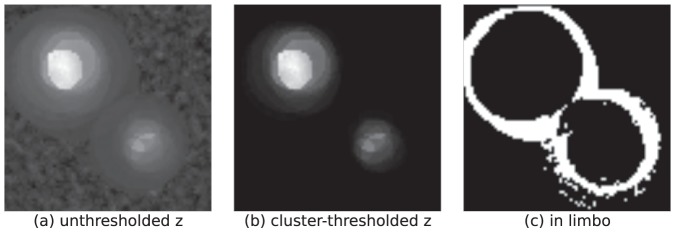
Simulated fMRI-data of multiple subjects. In all subjects two regions are differentially activated across conditions, but the precise shape, location, and effect size varies. The unthresholded z-map (a) shows large rings of overlapping individual regions of activation. In the thresholded map (b) only a small subset of these rings remains marked as significantly activated. The in limbo map (c) shows that these outer rings are not significantly less activated than some of the least-significantly significantly activated voxel in the inner regions.

The in limbo concept is illustrated in Fig. 2: the green regions do not differ significantly from zero, but neither do they differ significantly from the least-significantly activated region 5. Figs. 3 and 4 show examples of in limbo regions in a simulated data SPM (for details see the section *Simulation Studies*).

Our in limbo idea can be implemented in several ways. For concreteness, we focus here on an intuitive method that consists of four main steps:

1. For every voxel in the brain, the size of the contrast of interest and corresponding variance is estimated using the General Linear Model and sandwich variance estimators [9].
2. The resulting statistical parametric map is thresholded using cNote that, to be able to choose a comparison voxel, of course there should be a significant effect of the task to begin with. If there are no significantly activated areas, there can be no regions that are in limbo.luster-based methods.
3. The voxel with the lowest t-value that is considered significantly different across conditions is selected as a comparison voxel.
4. The size of the contrast in voxels that were found not to be significantly different is compared to the size of the contrast in the comparison voxel. Non-significant voxels of which the contrast cannot be considered statistically different from the comparison voxel are considered in limbo. To test for this difference efficiently, the covariance between the tested voxels and the comparison voxel should be estimated and taken into account.

These steps will now be described in more detail. They have been implemented as an automated pipeline using the NiPype-framework [10] and can be downloaded from GitHub (https://github.com/Gilles86/in_limbo).

#### Step 1: Estimate Contrasts and Corresponding Variances Over The Conditions

By far the most popular method in fMRI-analysis is the so-called mass univariate general linear model (GLM) approach. The GLM also forms the basis of the in limbo approach outlined here. In the GLM approach, for every voxel 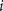, a set of 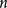 regressors 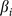 is fitted against the voxel activation time course 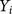 over 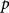 time points, using a 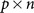 design matrix 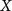. This design matrix is usually identical for all voxels. Each regressor describes the expected change in the BOLD signal as a result of one of the individual experimental conditions. Nuisance regressors such as head movement or linear scanner drift can also be included to remove unwanted noise and increase statistical power [11]. Given the estimators of the experimental regressor and their variances, it is possible to use inferential statistics to answer, for example, the question whether in a certain voxel the BOLD-response was significantly larger during condition 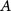 than during condition 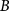.

The expected hemodynamical responses for the different experimental conditions are constructed by convolving a hemodynamic response function (HRF) with a model of the expected neural responses. These neural responses are usually modeled as a boxcar function of a few seconds, starting at the onset of a trial or stimulus. The columns of the design matrix 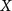 correspond to the experimental conditions and contain the expected hemodynamical responses for these conditions. This results in the following General Linear Model [12]:



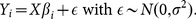
(1)



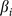
 can now be estimated by minimizing the error term using Ordinary Least Squares (OLS):



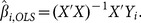
(2)


The variance matrix 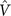 can be estimated by using the sum of the squared residuals, 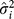:



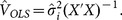
(3)


This simple approach has two limitations: (1) the errors in the residuals cannot be assumed to be independent, because there is autocorrelation in the signal [13], and (2) the model for the hemodynamic response is misspecified, because it is known to vary considerably across both individuals and brain areas [14]. As a consequence, variance estimates often will be too low, increasing the risk of Type I errors.

An often-used solution to these problems is to model and then remove the autocorrelation of the signal (prewhitening). This can for example be done by using an autoregressive model (AR). In that case the design matrix is first fitted to the data using the OLS approach. Subsequently, an AR model is fitted to the residuals. This procedure yields a model of the autocorrelation structure 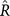. This AR model is then used in a generalized least squares (GLS) fit, where the errors that can be explained by the covariance structure 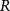 are removed, before the actual 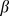-parameters are estimated. 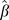 can then be estimated using



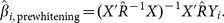
(4)


The variance can be estimated using



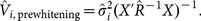
(5)


Unfortunately, it can be difficult to find an unbiased estimate of the autocorrelation structure 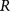 and the models that are used are even known to be incorrect [9], [14]. An alternative approach, one that we promote here, is to use the sandwich variance estimator. First suggested for use in fMRI by [9], this estimator is unbiased and robust against both autocorrelation and model misspecification. The key difference with traditional approaches is that the data and design matrix are divided up in 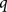 different blocks of replications 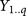 and 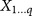. Note that these replications do not need to have identical design matrices 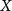, but the replicatons do have to be of the same length. The replications can represent individual runs or trials, but this is not a requirement. In our implementation, every functional run is divided up in 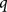 “subruns”, where 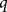 equals the square root of the number of time points in the run. Hence, there are q design matrices of 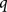 time points.

When using the sandwich estimator, the 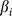-parameters are estimated by



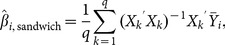
(6)


where 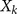 is the design matrix of the 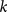-th replication and 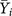 is the mean time course of all replications in voxel 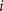. The variance matrix is given by the sandwich formula:



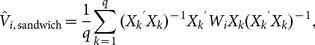
(7)


where 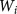 is an estimate of the covariance structure of the residuals in voxel 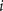, 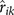:



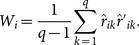
(8)


Contrasts of the different regressors can now be used to test for any differences in BOLD-signal between conditions, 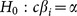 versus 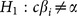. The contrast 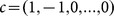 represents, for example, the difference in BOLD-signal between the first (e.g., task) and second (e.g., rest) experimental regressor, which can be tested to differ from zero by using 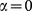.

Importantly, the sandwich estimator can also be used to estimate the amount of covariance between two voxels 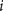 and 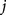, within a single contrast 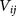:



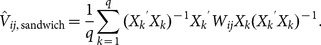
(9)


Here 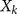 is the design matrix of replication 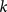 and



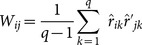
(10)


describes the covariance structure of the residuals in voxel 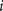 and 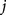. 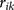 is the residual of voxel 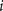 at replication 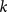, 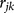 is the residual of voxel 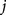 at replication 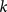. The covariance estimator is important for the in limbo approach presented here, because a test for a difference between two voxels ideally takes their covariance into account.

#### Step 2: Thresholding of Statistical Maps

After the regression parameters 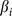 and their corresponding variances are fit, the next step is to threshold the resulting z-map of the contrast of interest. This can be done either at the level of an individual subject, or at the level of multiple subjects. In the second case, the individual contrast maps first have to be registered to a common space and combined in a second model to yield a level-2 z-map [11], [15].

For statistical inference on a very high number of correlated test statistics, such as in an fMRI z-map, a balance must be found between power and sensitivity in the multiple comparison problem. A standard Bonferroni correction, where the 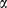-value is simply divided by the number of comparisons, is inappropriately conservative, because the test statistics are correlated. Many alternative approaches to the Bonferroni approach have been proposed [16]. Here we use the standard clustering algorithm implemented in the FSL suite (Functional MRI of the Brain Software Library; www.fmrib.ox.ac.ukfsl), which first thresholds the entire image at some z-value (for example z  =  2.3, corresponding to an uncorrected p <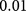 threshold) and then the probability of clusters of above-threshold voxels to occur under the null hypothesis is estimated using Gaussian random field theory (GRF). This correction takes into account both the total number of tested voxels (comparisons), the number of above-threshold clusters, as well as the correlations amongst neighboring voxels (i.e., the smoothness of the statistical map; [17]).

#### Step 3: Determine The Comparison Voxel

After the z-map has been thresholded, a comparison voxel 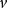 can be selected. Here we select the voxel with the lowest test statistic 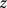 that still survived the thresholding procedure. This voxel 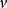 can be described colloquially as “the least significantly activated voxel” of that particular contrast. Other selection criteria for the comparison voxel are possible. For example, one could take the average contrast size in the least-significant cluster. We chose these particular criteria for selecting the comparison voxel, because they are easy to implement and interpret.

Note that, to be able to choose a comparison voxel, of course there should be a significant effect of the task to begin with. If there are no significantly activated areas, there can be no regions that are in limbo.

#### Step 4: Determine In Limbo Regions

For this step we distinguish between analyses for single subjects and for multiple subjects.

#### Single Subject

For every voxel 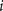 that was not significantly activated in the contrast of interest, a test is performed on whether the size of this contrast is significantly smaller than the size of the same contrast in the comparison voxel 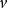.

This is done by taking the difference between the size of the contrast in the two voxels and dividing it by a pooled variance term, including the variance of both voxel contrasts, as well as the covariance between these two. Comparing voxel 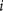 and to voxel 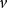 the following t-statistic is used:



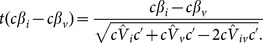
(11)


This t-statistic can be used for regular inference testing. The null hypothesis is that there is no difference between the size of the contrast in the non-activated voxel 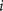 and the same contrast in the least-significantly activated comparison voxel 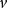.

If this null hypothesis cannot be rejected, voxel 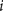 is not activated less than the compariso voxel 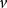, in addition to not being significantly activated in the first place: in other words, voxel 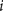 is in limbo. In contrast, if the null hypothesis can be rejected, the voxel is significantly activated less than the significantly activated areas, and claims about selective activation are warranted.

#### Multiple Subjects

In the case of multiple subjects, the number of data points in the analysis corresponds to the number of subjects, 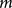. Again, for all non-significantly activated voxels, the hypothesis to test is whether the size of their contrast differs significantly from that of the least significantly activated comparison voxel.

To carry out this analysis, we use a weighted least squares (WLS) approach. We do this to increase power by weighting the individual subject by the precision (i.e., the inverse of the variance) of the estimate of the difference between the size of the contrast in the voxel of interest 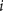 and the comparison voxel 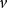.

For every non-significantly activated voxel 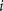, a 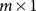 vector 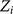 is set up, where every element represents one of the 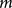 subjects. The 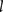
th element of this vector contains the difference between the size of the contrast in that specific voxel in subject 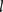 (i.e., 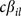) and the size of the same contrast in the comparison voxel 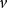 (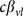):



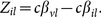
(12)


In addition, for every non-significantly activated voxel, we set up an identical 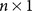 design matrix 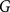, filled with only ones and a 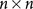 diagonal weight matrix 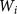. The diagonal of 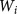 contains the inverse of the standard deviations (i.e., square root of the variance) of the estimate of the difference between the comparison voxel and that particular voxel, 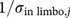:
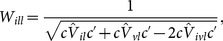
(13)


where 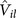 is the variance of voxel 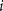 in subject 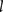 and 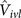 is the covariance between voxel 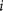 and comparison voxel 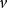 in subject 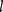 as given in Equations 7 and 9.

Finally, we solve the following system of equations:



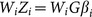
(14)


using
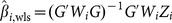
(15)


and
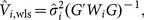
(16)


where 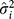 are the summed squared residuals in voxel 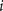. Solving these systems yields both an estimator of the difference in the size of the contrast in this voxel 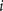 and the comparison voxel 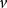 at the level of the group of subjects, 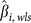, as well as an estimator of the variance of this difference, 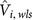. These estimates can be used to calculate a t-value to test the hypothesis that there is no difference between the sizes of the contrast in the two voxels, similar to Equation 11. If this hypothesis cannot be rejected at some significance threshold (here we use 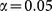), the size of the contrast in voxel 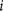 is not significantly different from the same contrast in the comparison voxel 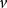 and we conclude that voxel 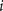 is in limbo.

#### Multiple Comparisons Correction

Similar to the standard multiple comparisons corrections on the original contrast, a multiple comparisons correction procedure could also be used for the final t-test that compares the contrast size in all non-significantly activated voxels against the same contrast in the comparison voxel. Procedures such as false discovery rate (FDR) or Gaussian random field theory are both feasible. An FDR approach might be more appropriate than a GRF approach, because it is unlikely that the in limbo landscape of t-values behaves like a Gaussian random field. Here we opted not to apply any multiple comparisons correction to the in limbo t-values at all. Application of a multiple comparisons procedure will increase the number of voxels that are in limbo. The current procedure, without any multiple comparisons correction thus results in a minimum number of voxels that are in limbo.

## Results

### Simulation Studies

Here we present results from two simulation studies that show the effect and usefulness of the in limbo approach. The simulation studies in this section illustrate potential scenarios in which an in limbo approach offers additional information over and above conventional approaches. The data in these studies were simulated using the neuRosim package from [18] and analyzed using custom-built Python scripts.

#### A Single Subject

In this scenario, a single synthetic subject with a square, 2D-brain employs two brain regions that are differentially activated compared to the rest of the brain: region A and B.

As shown in Fig. 3a, Region A is most strongly activated and, consequently, its task-induced activation survives the cluster-based thresholding. However, the weaker task-induced activation in region 2 does not survive the thresholding (see Fig. 3b).

From the original SPM, one might be inclined to draw the conclusion that region A is selectively activated in the task, in the sense that its activation is substantially larger than that in other regions (such as region B).

However, this conclusion is based on the imager's fallacy: to test the hypothesis of selective activation, one needs to test the difference between regions directly. After applying the limbo approach it is evident from Fig. 3c why the above conclusion was fallacious: for the main part of brain region B, the increase in activation during the task is not significantly less than the least significant increase in activation in region A.

#### Multiple Subjects

In a second simulation study we generated data from an fMRI experiment with multiple subjects. In the ground truth of this simulation, two regions show increased activation in the experimental condition in all subjects. However, the effects sizes and the precise location and size of these regions slightly differed across subjects. When applying the in limbo approach to these data (see Fig. 4), one can see that both regions are clearly visible in the thresholded level-2 z-map, but the in limbo map shows that the precise location of these regions is, on a group-level, more uncertain and less focused than the thresholded map might suggest.

### Real-world Example

The in limbo approach was also applied to real-world fMRI data, taken from [19] (this dataset is fully available upon request. Please email the original study's first author, Birte Forstmann (buforstmann@gmail.com), or the first author of this study (gilles.de.hollander@gmail.com)). In this study, subjects performed a forced-choice two-alternative random dot motion (RDM) task and were cued to stress either the speed or accuracy of their decision. The main finding of the study was that both the right striatum and presupplementary motor area (preSMA) show higher activation when subjects were instructed to stress the speed of their decision as compared to when they stressed the accuracy of their decission.

Re-analysis of the data using a contrast of “speed condition – accuracy condition" and a general linear model using a sandwich estimator showed similar activation patterns as reported in the original study. Right striatum and preSMA were increasingly activated in the speed-stressed condition as compared to the accuracy-stressed condition. A similar speed stress-related increase was found in the bilateral insula and dorsolateral prefrontal cortex. The latter brain regions were not found in the original study; this is probably due to higher thresholds and the lack of a conjunction-analysis here.

**Figure 5:**
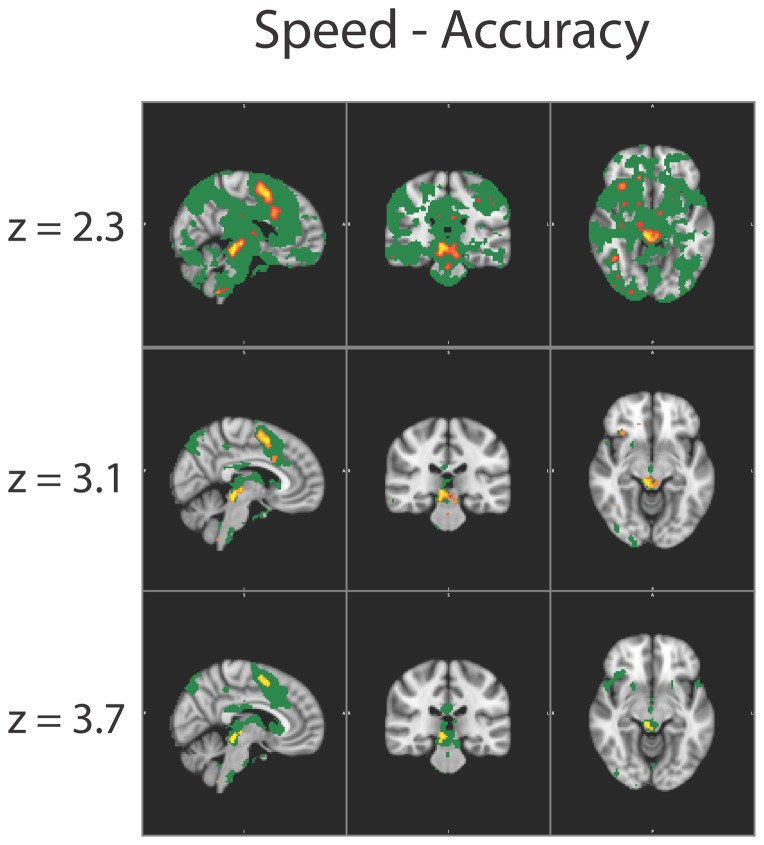
Results of the in limbo approach on actual fMRI-data from Forstmann et al. [19], using different z-thresholds of 2.3, 3.1 and 3.7, corresponding to uncorrected single-sided *α*-values of p <0.01, p <0.001 and p <0.0001. After this initial thresholding procedure, these surviving areas were corrected at the cluster-level using Gaussian random field theory. The in limbo areas are not significantly different from the least-significantly activated brain area, according to a t-test with an alpha-level of p<0.05, uncorrected for multiple comparisons. Legend: *Yellow-red* areas are significantly activated, *green* areas are in limbo. Note how with a rather liberal threshold of 2.3 almost the entire brain is in limbo and increasing the z-threshold from 3.1 to 3.7 does not change much in volume of brain that is in limbo. Brains are in MNI-152 space with slices at X = 42, Y = 65, Z = 65.

When applying the in limbo approach to these data, it becomes apparent that experimental effects are less specific than the original SPM may suggest. Especially at a liberal, but common z-threshold of 2.3, a large part of the brain is in limbo, as can be seen in the large green areas in Fig. 5 and Table 1. At a slightly higher z-threshold (3.1, corresponding to p <0.001 uncorrected), both the volume of activated (orange) areas as the volume of in limbo (green) areas decreases. Increasing the z-threshold further does not reduce the size of areas in limbo. At a z-threshold of 4.1, the size of areas of in limbo area even increases slightly. Further analyses revealed that when the threshold was set at this value of 4.1, a very different comparison voxel was chosen than those in the other threshold settings. This voxel has, consequently, a different covariance pattern which resulted in lower t-values in the in limbo tests, increasing the area that was labeled in limbo.

**Table 1:** Size of areas in limbo for different thresholds.

z-threshold	Volume above threshold (cm^3^)	Volume in limbo (cm^3^)
2.3	60	813
3.1	14	61
3.7	5	93
4.1	1	188

The amount of area that is labeled in limbo for different z-thresholds applied to the dataset of Forstmann et al., [19].

The regions that are marked in limbo are sensible, as they encompass both (1) distinct networks that are known to have relatively high variance (e.g., regions in the midbrain), as well as (2) regions that are significantly activated at lower z-thresholds or surround regions that are significantly activated. In sum, the in limbo approach seems to produce sensible results for real fMRI-data, complementing traditional SPMs with important additional information.

## Discussion

The in limbo approach presented here can be useful in different stages of a neuroimaging analysis. First and foremost, the in limbo approach can be used to warrant theoretically meaningful claims such as “region A was selectively involved in this task”. Secondly, the approach can be used to choose appropriate z-thresholds that are strict enough to ascertain that the effects in significantly activated regions are not similar in effect size to many other brain regions that are not significantly activated. Third, the approach can serve as a qualitative test to assess the extent to which a study is underpowered. When a large part of the brain is in limbo, one might be careful in drawing strong conclusions and consider conducting a follow-up experiment with more participants and/or trials.

The principle of the in limbo approach could also be applied to other neuroimaging analysis techniques such as the multivariate pattern searchlight approach [20]. This increasingly popular approach does not measure mean BOLD activity but instead quantifies classifier decoding accuracy for different regions in the brain. For this technique as well as for the more standard analysis techniques, substantive research questions may center on the specificity of task involvement across different brain regions – hence, the in limbo style of analysis is just as useful for the multivariate pattern searchlight approach as in univariate studies.

One could, in a similar vein, also apply the in limbo approach to statistical parametric mappings of other (structural) imaging modalities, such as diffusion weighted imaging (DWI) and voxel-based morphometry (VBM). These imaging techniques are also susceptible to the imager's fallacy. To give an example: a DWI study could show, at a first glance, that the white matter integrity of the tracts between two specific brain regions is reduced in a patient group as compared to a control group. However, analysis of a larger cohort may show that white matter integrity in this patient group is actually reduced in the entire brain, but the smaller study does not have enough power to pick up this global effect. An in limbo-like approach could protect against the premature conclusion as it may show, also in the underpowered study, that the reduction is not specific to only one white matter tract.

One may rally against the in limbo approach and argue that it is problematic to compare BOLD responses between brain regions that differ considerably in neurovascular coupling. However, this argument holds for neuroimaging analyses in general: most approaches compare the activation of different brain regions, albeit implicitly, as the occurrence of activity in a given brain region is only interesting in the absence of activation elsewhere. Our approach makes this comparison process explicit. Inference remains valid in our approach because we used the sandwich estimator which accounts for misspecification in the HRF. However, future work could further improve inference by explicitly accounting for neurovascular differences between regions. Work on this topic features in the literature on connectivity measures such as psychophysiological interaction analysis (PPI, [21]) and dynamic causal modelling (DCM, [22]).

It is important to stress that the proposed approach is not a way to find marginal or trending regions that might be involved in the task. The in limbo approach informs researchers about the specificity of the significant activation patterns by identifying the areas whose activation patterns are not significantly different. One should refrain from drawing any strong conclusions about the areas that are found to be in limbo.

Researchers should be cautious in how to visualize areas that are in limbo. Here we adopted a color scheme where the in limbo areas are labeled green, to emphasize that a large volume in the brain can be in limbo when liberal significance thresholds are used. Alternatively, one may color code not the in limbo areas, but the areas that are not significantly activated, nor in limbo. However, this color code may tempt researchers to falsely conclude that the colored areas are “significantly inactivated”. This conclusion is invalid in the frequentist framework employed here.

In the approach presented here, we chose to pick the least-significantly activated voxel as the comparison voxel for all not significantly activated regions. One could argue that this approach is conservative and one could instead select, as a comparison standard, for example, the median z-value of the significantly activated regions. Doing so will lead to a smaller volume of regions that are judged to be in limbo. However, the interpretation of the areas that are not in limbo then becomes less straightforward: they are significantly different from the median-z value, but not necessarily different from less-significantly activated areas. When one uses the least-significantly activated voxel as the comparison voxel, one knows for sure that the effect sizes in areas that are not in limbo are all significantly different from those in all significantly activated areas. Therefore, we favor the use of the least-significantly activated voxel as a comparison voxel.

In our approach we chose to use the sandwich variance estimator, because the in limbo test should take into account both the within-voxel variance and the across-voxel variance. Both variances are underestimated by OLS estimators, even when the data are prewhitened with an AR(1)-model, as this autocorrelation model is known to be incorrect [9], [14]. The sandwich approach is, however, rather uncommon and computationally intensive. A simpler and computationally less intensive approach is to conduct a standard level–2 analysis, find the least-significantly activated voxel, subtract its effect size from the individual contrasts (separately for each participant) and conduct the level 2-analysis again. We implemented such an analysis and found in limbo areas that were similar but slightly smaller than those identified by the current approach, especially at lower z-thresholds (See http://nbviewer.ipython.org/github/Gilles86/in_limbo/blob/master/notebooks/level2_simple.ipynb for code and more details). When one trusts the AR(1)-whitening procedure, a similar approach could be implemented that takes into account the covariance between voxels. The practical importance of any differences between the various methods remains an empirical question and awaits further research.

The attentive reader that takes a closer look at the in limbo areas at different thresholds in Fig. 5, will note that at higher thresholds, the areas that are in limbo highly overlap with significantly activated areas at slightly less-stringent thresholds, albeit they are not the same. One could ask why one should apply the in limbo approach and not just “lower the threshold”. The answer to this question lies in the interpretability of the results: when lowering the threshold, this will most likely done choosing an arbitrary amount. In contrast, the in limbo approach offers a theoretically founded, meaningful map of areas that are not significantly different from areas that differ from baseline.

To conclude: we presented a new statistical method to aid researchers in their fMRI analyses, by highlighting regions in which the experimental effect size is in limbo. Regions that are in limbo differ neither from baseline nor from regions that are significantly different from baseline. The in limbo approach helps reduce the impact of the imager's fallacy, allows researchers to choose sensible statistical thresholds, and promotes sound inferences.
